# Correction: Combating drug-resistant bacteria with sulfonium cationic poly(methionine)

**DOI:** 10.1039/d3ra90121a

**Published:** 2023-12-14

**Authors:** Lizhong Zhu, Jie Li, Weiwei Huan

**Affiliations:** a Zhejiang Provincial Key Laboratory of Chemical Utilization of Forestry Biomass, College of Chemistry and Materials Engineering, Zhejiang A&F University Hangzhou Zhejiang 311300 China

## Abstract

Correction for ‘Combating drug-resistant bacteria with sulfonium cationic poly(methionine)’ by Lizhong Zhu *et al.*, *RSC Adv.*, 2023, **13**, 27608–27612, https://doi.org/10.1039/D3RA03925K.

The authors regret the omission of the funding acknowledgements in the original article. These acknowledgements are given here.

This work was financially supported by the Key Research Program of Zhejiang Province (2021C02041 and 2021C02013), the National Natural Science Foundation of China (32201238), the “Sannongjiufang” Research Joint Project of Zhejiang Province in 2022 (2022SNJF059) and the “Sannongliufang” Research Joint Project of Zhejiang Province in 2021 (2021SNLF008).

The Royal Society of Chemistry apologises for these errors and any consequent inconvenience to authors and readers.

## Supplementary Material

